# Respirable crystalline silica and lung cancer in community-based studies: impact of job-exposure matrix specifications on exposure–response relationships

**DOI:** 10.5271/sjweh.4140

**Published:** 2024-04-01

**Authors:** Johan Ohlander, Hans Kromhout, Roel Vermeulen, Lützen Portengen, Benjamin Kendzia, Barbara Savary, Domenico Cavallo, Andrea Cattaneo, Enrica Migliori, Lorenzo Richiardi, Nils Plato, Heinz-Erich Wichmann, Stefan Karrasch, Dario Consonni, Maria Teresa Landi, Neil E Caporaso, Jack Siemiatycki, Per Gustavsson, Karl-Heinz Jöckel, Wolfgang Ahrens, Hermann Pohlabeln, Guillermo Fernández-Tardón, David Zaridze, Jolanta Lissowska, Beata Swiatkowska, John K Field, John R McLaughlin, Paul A Demers, Tamas Pandics, Francesco Forastiere, Eleonora Fabianova, Miriam Schejbalova, Lenka Foretova, Vladimir Janout, Dana Mates, Christine Barul, Thomas Brüning, Thomas Behrens, Kurt Straif, Joachim Schüz, Ann Olsson, Susan Peters

**Affiliations:** 1 Institute for Risk Assessment Sciences, Utrecht University, Utrecht, the Netherlands.; 2 Julius Center for Health Sciences and Primary Care, University Medical Center, Utrecht, the Netherlands.; 3 Institute for Prevention and Occupational Medicine of the German Social Accident Insurance – Institute of the Ruhr-Universität Bochum, Germany.; 4 Institut National de Recherche et de Sécurité, Vandoeuvre lès Nancy, France.; 5 Department of Chemical and Environmental Sciences, Università degli Studi dell’Insubria, Como, Italy.; 6 Department of Occupational and Environmental Health, Università degli Studi di Milano, Milan, Italy.; 7 Cancer Epidemiology Unit, CeRMS and University of Turin, Italy.; 8 Institute of Environmental Medicine, Karolinska Institutet, Stockholm, Sweden.; 9 Institute of Epidemiology, Helmholtz Zentrum München-German Research Center for Environmental Health, Neuherberg, Germany.; 10 Institut für Medizinische Informatik Biometrie Epidemiologie, Ludwig Maximilians University, Munich, Germany.; 11 Institute and Clinic for Occupational, Social and Environmental Medicine, University Hospital LMU Munich; Comprehensive Pneumology Center Munich (CPC-M), Member of the German Center for Lung Research (DZL), Munich, Germany.; 12 Unit of Epidemiology, Fondazione IRCCS Ca’ Granda – Ospedale Maggiore Policlinico, Milan, Italy.; 13 National Cancer Institute, Bethesda, USA.; 14 Research Centre of University of Montreal Hospital Centre, University of Montreal, Canada.; 15 Institute for Medical Informatics, Biometry and Epidemiology, University of Duisburg-Essen, Germany.; 16 Bremen Institute for Prevention Research and Social Medicine, Germany.; 17 Instituto Universitario de Oncología, Universidad de Oviedo, Oviedo, Spain.; 18 Russian Cancer Research Centre, Moscow, Russia.; 19 The M Sklodowska-Curie Cancer Center and Institute of Oncology, Warsaw, Poland.; 20 The Nofer Institute of Occupational Medicine, Lodz, Poland.; 21 Roy Castle Lung Cancer Research Programme, The University of Liverpool Cancer Research Centre, Department of Molecular and Clinical Cancer Medicine, Institute of Translational Medicine, University of Liverpool. UK.; 22 Occupational Cancer Research Centre, Toronto, Canada.; 23 National Institute of Environment Health, Budapest, Hungary.; 24 Department of Epidemiology, Regional Health Service, Rome, Italy.; 25 Regional Authority of Public Health, Banska Bystrica, Slovakia.; 26 Institute of Hygiene and Epidemiology, 1st Faculty of Medicine, Charles University, Prague, Czech Republic.; 27 Masaryk Memorial Cancer Institute, Brno, Czech Republic.; 28 Palacky University, Faculty of Medicine, Olomouc, Czech Republic.; 29 Institute of Public Health, Bucharest, Romania.; 30 Institut de recherche en santé, environnement et travail, Guadeloupe, France.; 31 ISglobal, Barcelona, Spain and Boston College, USA.; 32 International Agency for Research on Cancer, Lyon, France.

**Keywords:** case-control study, general population, JEM, lung neoplasm, quantitative exposure assessment, respirable quartz exposure

## Abstract

**Objectives:**

The quantitative job-exposure matrix SYN-JEM consists of various dimensions: job-specific estimates, region-specific estimates, and prior expert ratings of jobs by the semi-quantitative DOM-JEM. We analyzed the effect of different JEM dimensions on the exposure–response relationships between occupational silica exposure and lung cancer risk to investigate how these variations influence estimates of exposure by a quantitative JEM and associated health endpoints.

**Methods:**

Using SYN-JEM, and alternative SYN-JEM specifications with varying dimensions included, cumulative silica exposure estimates were assigned to 16 901 lung cancer cases and 20 965 controls pooled from 14 international community-based case-control studies. Exposure–response relationships based on SYN-JEM and alternative SYN-JEM specifications were analyzed using regression analyses (by quartiles and log-transformed continuous silica exposure) and generalized additive models (GAM), adjusted for age, sex, study, cigarette pack-years, time since quitting smoking, and ever employment in occupations with established lung cancer risk.

**Results:**

SYN-JEM and alternative specifications generated overall elevated and similar lung cancer odds ratios ranging from 1.13 (1^st^ quartile) to 1.50 (4^th^ quartile). In the categorical and log-linear analyses SYN-JEM with all dimensions included yielded the best model fit, and exclusion of job-specific estimates from SYN-JEM yielded the poorest model fit. Additionally, GAM showed the poorest model fit when excluding job-specific estimates.

**Conclusion:**

The established exposure–response relationship between occupational silica exposure and lung cancer was marginally influenced by varying the dimensions of SYN-JEM. Optimized modelling of exposure–response relationships will be obtained when incorporating all relevant dimensions, namely prior rating, job, time, and region. Quantitative job-specific estimates appeared to be the most prominent dimension for this general population JEM.

Traditionally, the most detailed and often quantitative exposure assessment and assignment in occupational epidemiology has been obtained through monitoring results in specific industries. Notably, the established occupational silica-lung cancer relationship is primarily based on the modelling and synthesis of risk estimates obtained in industry-based studies (1, 2). However, community-based case–control studies frequently yield larger sample sizes (and more exposed cases), more complete lifetime job histories, and more comprehensive information on crucial confounding variables like eg, smoking habits, albeit the exposure assessment and assignment for such studies is typically semi-quantitative based on self-reported exposures or general population job-exposure matrices (JEM) (3–5). The advantageous properties of a community-based study can still be utilized in the occupational exposure assessment process through applying a quantitative JEM, where job histories and detailed measurement data are combined, enabling the generation of quantified exposure estimates and derivation of associated exposure–response relationships (6).

Approximately a decade ago, we introduced SYN-JEM, a quantitative JEM designed for community-based epidemiological studies, developed within the SYNERGY project on lung cancer (7–9). SYN-JEM yields exposure estimates through the application of different dimensions: job-specific estimates, region-specific estimates, and prior expert ratings of jobs by the semi-quantitative job-exposure matrix DOM-JEM (10). The expert ratings serve as priors in the statistical model and are calibrated by personal quantitative silica measurements collated in the international exposure database ExpoSYN (9). The development of SYN-JEM and analogous quantitative JEM, eg, for benzene, noise, and daytime light exposure (11–13), constitutes a significant advancement in exposure assessment methodology and has improved and facilitated the assessment of quantitative exposure levels in community-based settings, achieving a level of quality comparable to that of industry-based studies (6).

Most population-based studies, however, use less sophisticated JEM based on fewer dimensions. Thus far, no study investigated how differences in the dimensions applied in a quantitative JEM influence occupational exposure estimates and associated risk of different health endpoints. We therefore investigated these interdependencies through analyzing the established exposure–response relationship between occupational silica exposure and lung cancer risk in the SYNERGY population, using the original SYN-JEM and alternative specifications of SYN-JEM with varying dimensions included.

## Methods

### Study population

The SYNERGY population comprises pooled data of 17 705 lung cancer cases and 21 813 controls originating from 14 population- or hospital-based case–control studies conducted between 1985 and 2010 in 13 European countries and Canada. Full details of the SYNERGY project (http://synergy.iarc.fr) and data collection is presented elsewhere (7). For all subjects, detailed lifetime occupational and smoking history was available. For MORGEN, which was the only nested case–control study, smoking and occupational information was lacking for the time interval between enrollment and diagnosis or end of follow-up (mean interval <10 years). Ethical approvals were obtained in accordance with legislation in each country and by the IARC institutional review board.

### Exposure assessment using SYN-JEM

SYN-JEM is based on a semi-quantitative JEM called DOM-JEM (10), which assigns job-specific silica exposure ratings based on a combination of exposure probability and intensity scores (exposure ratings: no, low and high) (7, 8, 14, 15) for each International Standard Classification of Occupations, version 1968, (ISCO-68) job code (16). Full details on SYN-JEM have been published earlier (7), and more details are provided in the supplementary material (www.sjweh.fi/article/4140), file 1. SYN-JEM yields exposure estimates through the application of different dimensions: job-specific estimates, region-specific estimates, and the DOM-JEM ratings. In the underlying exposure estimation model for SYN-JEM, the DOM-JEM ratings serve as priors (hereafter DOM-JEM prior), which are calibrated through the inclusion of personal quantitative silica measurements collated in the international exposure database ExpoSYN (9) into the model. The modeling enables SYN-JEM to generate job-, region-, and time-specific quantitative exposure levels, standardized to an eight-hour work shift and representative work situations (7).

Furthermore, SYN-JEM assigns 0 mg/m^3^ to jobs that DOM-JEM rates as nonexposed (nonexposed override). Thus, the nonexposed override prevents that nonrepresentative non-zero exposure measurements in ExpoSYN, such as measurements made under extraordinary conditions and/or measurements that are solely representative for a small percentage of participants of a job group, are used to assign exposure for an entire a priori nonexposed job group.

Even with relatively few measurements, the application of SYN-JEM based on the calibrated DOM-JEM priors will still generate quantitative exposure–response relationships as previously demonstrated by Olsson et al (17) for the association between exposure to polycyclic aromatic hydrocarbons and lung cancer.

### Exposure assessment using SYN-JEM and alternative SYN-JEM specifications

Each study participant’s silica exposure was estimated using the original SYN-JEM (JEM 1), and alternative SYN-JEM specifications (JEM 2–5) in which the dimensions included for exposure estimation were varied accordingly: (i) JEM 1 – the original SYN-JEM – exposure estimates are based on all SYN-JEM dimensions (region-specific estimates, job-specific estimates, and the DOM-JEM prior); (ii) JEM 2 – SYN-JEM without region-specific estimates – all regions obtained the same job-specific estimates; (iii) JEM 3 – SYN-JEM without job-specific estimates – job assignments were based on the calibrated DOM-JEM prior and region-specific estimates; (iv) JEM 4 – SYN-JEM without either region- or job-specific estimates – only the calibrated DOM-JEM prior was assigned, with the same estimates for all regions; (v) JEM 5 – SYN-JEM without the DOM-JEM prior – estimates were entirely based on measurements (apart from when jobs were considered a priori nonexposed, in which case the nonexposed override was applied).

An overall downward time trend across regions and industries/jobs was applied in all SYN-JEM specifications. Details of SYN-JEM and alternative SYN-JEM specifications are further described in the supplementary file 1.

### Cumulative exposure assessment

Study participants’ cumulative exposure levels were estimated using the original SYN-JEM (JEM 1) and alternative SYN-JEM specifications (JEM 2–5), respectively. Cumulative exposure in mg/m^3^-years was estimated through assigning jobs held in each working year of each study participant an exposure level through linkage with SYN-JEM or its alternative specifications.

### Statistical analyses

The effect of each SYN-JEM dimension on categorical exposure–response relationships was analyzed using logistic regression models predicting lung cancer risk associated with quartiles of cumulative silica exposure (quartiles based on the JEM specific exposure distributions among controls) as per JEM 1–5. Further, using generalized additive models (GAM) we explored the shape of the exposure–response relationship between occupational silica exposure and lung cancer risk through generating exposure–response curves based on JEM 1–5, respectively. Estimated silica exposure was modelled as the natural log of cumulative silica exposure; nonexposed workers were assigned an arbitrary value of ^2^/3 of the lowest value among the exposed based on the distribution of JEM 1 (0.0036 mg/m^3^). The number of basis functions for all GAM models was set to seven (k=7), which sufficiently allowed for non-linear exposure–response relationships. We classified degree of nonlinearity in the GAM based on the effective degrees of freedom (EDF): EDF=1, linear relationship; EDF>1 and ≤2, weakly non-linear relationship; EDF>2, highly non-linear relationship (18). Log-linear regression models were used to analyze the continuous exposure–response between log-cumulative silica exposure as per JEM 1-5 and lung cancer risk. No lag-time for silica exposure was applied to any of the models, as zero lag yielded the best model fit in SYNERGY (6).

For all statistical modelling, adjustments were made for age group (<45; 45–49; 50–54; 55–59; 60–64; 65–69; 70–74; >74 years), sex, study, tobacco smoking [log(cigarette pack-years+1)], time since smoking cessation (current; cessation >0–7, 8–15, 16–25, >25 years before interview/diagnosis; and never smokers), and ever employment in a ‘list A job’, ie, occupations and industries known to present an excess risk of lung cancer (19, 20). Current smoking was defined as having smoked ≥1 cigarette per day for ≥1 year and included having stopped smoking in the last 2 years before the diagnosis or interview. Cigarette pack-years were calculated accordingly: ∑ duration × average intensity per day/20. The Akaike Information Criterion (AIC) was used to compare model fit. All analyses were performed in R (version 3.6.3). GAM analyses were made using the R-package ‘mgcv’.

## Results

Having excluded workers with incomplete data on covariates (N=804 cases, 848 controls), the study population consisted of 37 866 participants [16 901 cases and 20 965 controls, as per Ge and colleagues (6)]. The mean age was 61.7 years, and 79% were men (table 1).

**Table 1:** Population characteristics of the 14 SYNERGY pooled lung cancer case-control studies. [SD=standard deviation].

		Overall				Cases				Controls		
		N	%	Mean (SD)		N	%	Mean (SD)		N	%	Mean (SD)
		37 866	100			16 901	45			20 965	55	
Sex	Male	30 056	79			13 621	80			16 503	78	
	Female	7810	21			3301	20			4534	22	
Age				61.7 9.6				62.0 9.2			9.9	61.5 (9.9)
Smoking status	Never	8522	23			1369	8			7153	34	
	Former	13 625	36			5432	32			8220	39	
	Current	15 692	41			10 100	60			5592	27	
Study	AUT-Munich (Germany)	6429	17			3180	19			3249	16	
	CAPUA (Spain)	1071	3			559	3			512	2	
	EAGLE (Italy)	3973	11			1908	11			2065	10	
	HdA (Germany)	2006	5			1004	6			1002	5	
	ICARE (France)	6188	16			2739	16			3449	17	
	INCO (Czech Rep.)	756	2			304	2			452	2	
	INCO (Hungary)	696	2			391	2			305	2	
	INCO (Poland)	1628	4			793	5			835	4	
	INCO (Romania)	404	1			179	1			225	1	
	INCO (Russia)	1179	3			599	4			580	3	
	INCO (Slovakia)	630	2			345	2			285	1	
	INCO (UK)	1357	4			441	3			916	4	
	LUCA (France)	562	2			280	2			282	1	
	LUCAS (Sweden)	3321	9			1014	6			2307	11	
	MONTREAL (Canada)	2681	7			1176	7			1505	7	
	MORGEN (NL)	158	0.5			43	0.3			115	0.5	
	PARIS (France)	396	1			169	1			227	1	
	ROME (Italy)	647	2			326	2			321	2	
	TURIN/VENETO (Italy)	2575	7			1086	6			1489	7	
	TORONTO (Canada)	1209	3			365	2			844	4	

In the categorical and linear exposure–response analyses, inclusion of all SYN-JEM dimensions (JEM 1) revealed the best model fit while exclusion of job-specific estimates (JEM 3) resulted in the poorest model fit (table 2). All quartiles of cumulative silica exposure based on JEM 1 showed significantly elevated risks of lung cancer with risk estimates ranging from 1.15 in the 1^st^ quartile (median cumulative exposure level: 0.21 mg/m^3^-years) to 1.45 in the 4^th^ quartile (median cumulative exposure level: 3.86 mg/m^3^-years) (table 3, table 4). All alternative SYN-JEM specifications showed statistically significant increased risks of lung cancer for all quartiles of exposure, with risk estimates ranging from 1.13 in the 1^st^ quartile (JEM 3 and 5) to 1.50 in the 4^th^ quartile (JEM 2) (table 3).

**Table 2:** Akaike Information Criterion for JEM 1-JEM5. [AIC=Akaike Information Criterion. Ntot=37866; GAM=generalized additive models]

AIC	JEM 1: SYN-JEM	JEM 2: SYN-JEM without region-specific estimates	JEM 3: SYN-JEM without job-specific estimates	JEM 4: SYN-JEM without region- or job-specific estimates	JEM 5: SYN-JEM without DOM-JEM prior (with nonexposed override)
Categorical	42197.64	42474.74	42477.61	42476.07	42475.39
Linear	42194.76	42471.34	42477.8	42473.28	42473.26
GAM	41993.52	41987.37	41995.66	41989.77	41991.73

**Table 3:** Adjusted ^a^ risk estimates of lung cancer associated with categorical (quartiles of silica exposure) and log-cumulative silica exposure, derived using the original SYN-JEM (JEM 1)), and different SYN-JEM specifications with varying dimensions of SYN-JEM included (JEM 2-5). [OR=odds ratio; 95% CI=95% confidence interval; AIC=Akaike Information Criterion. Ntot=37866]. Cut-offs for exposure quartiles are based on the JEM specific distribution of exposure among the controls.

	JEM 1: SYN-JEM		JEM 2: SYN-JEM without region-specific estimates		JEM 3: SYN-JEM without job-specific estimates		JEM 4: SYN-JEM without region- or job-specific estimates		JEM 5: SYN-JEM without DOM-JEM prior (with nonexposed override)
	OR (95% CI)		OR (95% CI)		OR (95% CI)		OR (95% CI)		OR (95% CI)
Cumulative silica exposure (mg/m^3^-years):									
Unexposed	1		1		1		1		1
1^st^ quartile	1.15 (1.04-1.27)		1.16 (1.05-1.28)		1.13 (1.02-1.25)		1.16 (1.05-1.27)		1.13 (1.02-1.25)
2^nd^ quartile	1.33 (1.21-1.47)		1.29 (1.17-1.42)		1.35 (1.23-1.49)		1.27 (1.15-1.41)		1.34 (1.22-1.48)
3^rd^ quartile	1.29 (1.17-1.42)		1.30 (1.18-1.43)		1.32 (1.20-1.46)		1.34 (1.21-1.47)		1.29 (1.17-1.42)
4^th^ quartile	1.45 (1.31-1.60) ^c^		1.50 (1.37-1.66) ^c^		1.42 (1.29-1.57) ^c^		1.48 (1.34-1.64) ^c^		1.46 (1.33-1.61) ^c^
Log-cumulative silica exposure: ^b^									
β-coefficient	1.049 (1.039-1.059)		1.048 (1.039-1.058)		1.050 (1.040-1.061)		1.050 (1.040-1.060)		1.047 (1.037-1.057)

^a^ Adjusted for age group, sex, study, cigarette pack-years, time-since-quitting smoking, and ever-employment in a ‘list A job’. ^b^ To enable log-transformation of exposure data generated with JEM 1-5, unexposed study participants were assigned two thirds of the lowest exposure value obtained using JEM 1 (0.0036 mg/m^3^-years). ^c^ p trend<0.001

**Table 4:** Exposure distributions for the original SYN-JEM (JEM 1), and different SYN-JEM specifications with varying dimensions of SYN-JEM included (JEM 2-5). Ntot=37866. Cut-offs for exposure quartiles are based on the JEM specific distribution of exposure among the controls.

Cumulative silica exposure (mg/m^3^-years)	JEM 1: SYN-JEM				JEM 2: SYN-JEM without region-specific estimates				JEM 3: SYN-JEM without job-specific estimates				JEM 4: SYN-JEM without region- or job-specific estimates				JEM 5: SYN-JEM without DOM-JEM prior (with nonexposed override)		
	Range	Median	N		Range	Median	N		Range	Median	N		Range	Median	N		Range	Median	N
1^st^ quartile	0.005-0.43	0.21	2234		0.009-0.56	0.32	2220		0.006-0.36	0.20	2229		0.009-0.50	0.25	2089		0.008-0.56	0.28	2202
2^nd^ quartile	0.43-1.12	0.72	2338		0.56-1.38	0.92	2372		0.36-0.94	0.61	2432		0.50-1.19	0.75	2505		0.56-1.43	0.96	2311
3^rd^ quartile	1.12-2.40	1.67	2355		1.38-3.10	2.11	2296		0.94-1.95	1.42	2342		1.19-2.56	1.75	2372		1.43-3.13	2.15	2362
4^th^ quartile	2.40-57.0	3.86	2484		3.11-24.2	4.68	2522		1.95-15.3	2.81	2408		2.56-9.77	3.59	2445		3.13-83.9	5.43	2536

**Figure 1:**
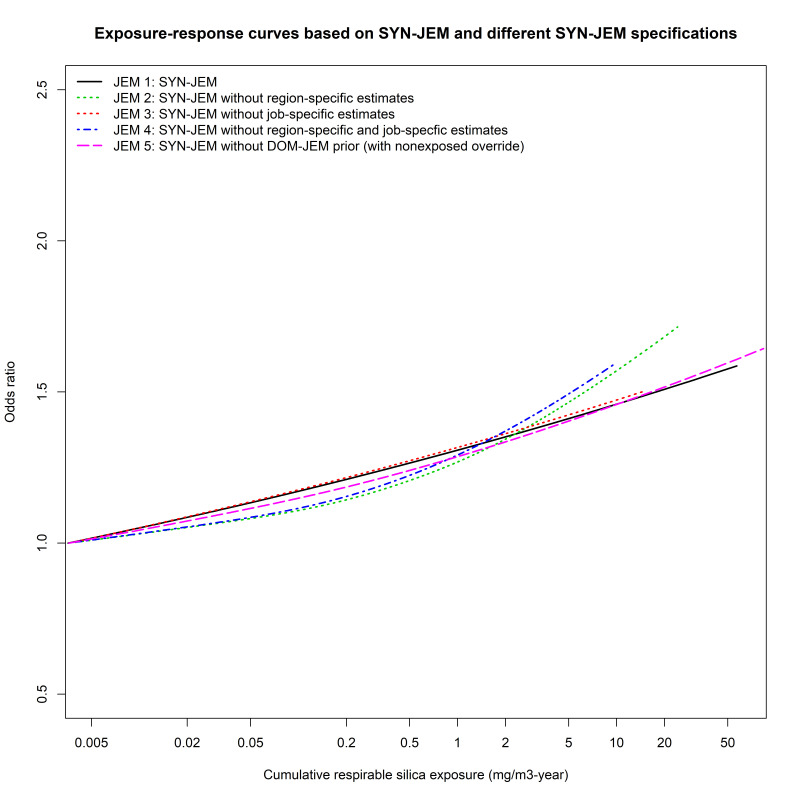
Exposure–response curves for cumulative silica exposure in relation to lung cancer risk based on general additive models (GAM), as per the original SYN-JEM (JEM 1), and alternative SYN-JEM specifications with varying dimensions of SYN-JEM included (JEM 2–5).

JEM 1 was associated with monotonical risk increases of 1.049 (95% CI 1.039–1.059) for every twofold increase in cumulative silica exposure (table 3). For JEM 2–5, the linear regression analyses showed similar statistically significant monotonically increasing lung cancer risks (β-coefficients varying between 1.047–1.050) (table 3). GAM analyses showed linear exposure–response relationships for JEM 1, 3 and 5, while for JEM 2 and 4 weak non-linear exposure–response relationships were apparent (table 3, figure 1). Based on the AIC, the best model fit in the GAM analyses was associated with removal of region-specific estimates (JEM 2), and the poorest model fit with removal of job-specific estimates (JEM 3) (table 2).

Supplementary figures S1–S4 show GAM generated exposure–response curves with 95% CI for JEM 1 compared with each of JEM 2–5.

## Discussion

We aimed to analyze the influence of applying different dimensions of a quantitative JEM on the established silica-lung cancer relationship. We demonstrated these interdependencies using SYN-JEM with varying dimensions applied on the pooled international community-based case–control study SYNERGY and found very similar exposure–response relationships. The original SYN-JEM with all dimensions included and the alternative SYN-JEM specifications showed significantly increased odds ratios ranging from 1.13 in the 1^st^ quartile to 1.50 in the 4^th^ quartile. In the categorical and linear analyses, the original SYN-JEM yielded the best model fit and SYN-JEM without job-specific estimates the poorest. The original SYN-JEM was associated with lung cancer risk estimates that increased monotonically with a factor of 1.05 (95% CI 1.04–1.06) for every twofold increase in cumulative silica exposure. Monotonically increasing lung cancer risks were also seen for the alternative JEM. The best model fit in the GAM analyses was found for the SYN-JEM specification without region-specific estimates, whereas the poorest fit was associated with SYN-JEM without job-specific estimates.

All analyzed SYN-JEM specifications showed similar lung cancer risk estimates per exposure quartile and in the continuous exposure–response analyses (table 3, figure 1), and would thus yield similar conclusions regarding the investigated exposure–response relationship. However, when varying the dimensions included in SYN-JEM and the method for modelling the exposure–response relationship (categorical, linear, GAM) informative patterns in terms of model fit and the shape of the exposure–response curves could be discerned. Removal of the job-specific estimates yielded the poorest model fit regarding the exposure–response curves in the categorical, linear and GAM analyses. Evidently, assignment of job-specific estimates efficiently improved the precision of the modeled exposure–response relationship between silica exposure and lung cancer risk and should thus be part of the quantitative JEM. When considering the possibilities of a non-linear exposure–response relationship between silica exposure and lung cancer risk by GAM modelling, exclusion of region-specific effects (JEM 2, JEM 4) yielded a slightly better model fit than remaining JEM. The associated weakly non-linear exposure–response is possibly partly a result of a shrunken exposure distribution resulting from not assigning participants region-specific effects, which we previously have shown to have an approximately 4.5-fold-range in exposure between regions (8). The slightly improved model fit when ignoring the region-specific estimates might partly be due to bias following insufficient adjustments made regarding the different measurement and analytical strategies applied in the participating countries. Additionally, as some countries yielded limited number of measurements, we categorized the regions a priori. Still, some regions comprise relatively few measurements (particularly Southern and Western Europe) and generated thus less precise region-specific estimates. Nevertheless, since regional differences in occupational exposures do exist (which also became apparent from our model), incorporation of region-specific estimates is preferred when such data is available. This is for example evident in the case of assigning UV exposure using quantitative JEM, where the exposure intensity indeed vary by geographic location. Thus, when applying an exposure assessment framework, such as that of SYN-JEM, one must incorporate expert knowledge and take into account the various particularities of each exposure of interest.

The original SYN-JEM yielded a monotonically increasing lung cancer risk estimate of 1.05 (95% CI 1.04–1.06) for every twofold increase in cumulative silica exposure, which is a less steep increase compared with the results of some previously published industry-based studies (1, 21). Moreover, the categorical results based on the original SYN-JEM generated a similar positive exposure–response trend compared with the same industry-based studies (1, 21), albeit with overall lower cumulative exposure levels (per exposure strata) and associated risk estimates. Compared with the recent meta-analysis by Shahbazi et al (2) of industry-based and community-based studies, the risk estimates per exposure strata were overall similar within SYNERGY (particularly <1.00 mg/m^3^-years), albeit Shahbazi et al reported lower risk estimates for the exposure range 1.00–1.99 mg/m^3^-years based on pooled estimates of an approximately equal number of community-based and industry-based studies, and slightly higher risk estimates for exposures above 6.00 mg/m^3^-years based on pooled estimates of mainly industry-based studies. The overall low exposure levels (particularly when compared with industry-based studies) assigned to the community-based SYNERGY population were expected, as jobs with relatively low exposures and short-term workers are present in general population studies, but not so in industry-based studies. Still, the median exposure levels in the 1^st^ and 2^nd^ exposure quartiles of the SYNERGY population (0.21 and 0.72 mg/m^3^-years, respectively) were associated with significantly increased lung cancer risks (OR≥1.15), which is comparable to the results of Shahbazi et al (2) who found a significantly increased lung cancer risk ratio of 1.14 for the lowest exposure range (0.00-0.49 mg/m^3^-years). In contrast, the meta-analysis by Lacasse et al (22) of mainly industry-based studies found significantly increased risks first at higher exposure levels, reporting a threshold value of 1.84 mg/m^3^-years.

Despite achieving the highest model precision when applying job-specific estimates, the current in-depth analyses of SYN-JEM and its dimensions have demonstrated that a quantitative JEM generates valid exposure–response relationships regardless the presence of (sufficient) measurement data to generate job specific and/or region-specific estimates. This methodological flexibility and associated relatively small trade-off in terms of precision of modelled exposure–response estimates makes quantitative JEM a yet more powerful exposure assessment tool, applicable to different types of community-based epidemiological studies with various types and quantities of data at hand.

We also show that quantitative JEM allow for the modeling of lung cancer risk estimates at relatively low cumulative exposure, present in community-based studies. Such detailed analyses of low levels of work-related silica exposure and associated lung cancer risks will inform the further need for preventive measures in the workplace. Only in the EU, approximately 400 000 incident lung cancers related to occupational crystalline silica exposure have been projected in the next five decades, even at an OEL of 0.2 mg/m^3^ (23). Accurate quantitative estimation of occupational exposures and corresponding disease risk estimates in community-based studies is key to enable the large-scale prevention required to mitigate and reduce the incidence of occupational diseases. Specifically, more accurate exposure estimates will help tailor and justify the development and implementation of more effective interventions to reduce chemical exposures in the workplace, which indeed are limited in number and quality (24).

The application and analysis of alternative SYN-JEM specifications with varying dimension of SYN-JEM included was made based on vast amounts of pooled international data on exposure, health outcome, and important confounders, which increases both the internal and external validity of our findings. Our methodology allowed us to evaluate potential bias in lung cancer risk estimates that several critical decisions related to the methodology of SYN-JEM might have introduced, which adds to the transparency and reproducibility of our findings, and further details the implications of applying SYN-JEM. Moreover, quantitative exposure–response analyses were made possible through the development of the ExpoSYN database, which with its >23 000 personal silica measurements originating from 13 European countries and Canada is truly unique in its kind. Nevertheless, the measurements were collated within different studies, over different time periods, and by different sampling strategies and sampling and analytical methods. Thus, the quality of the measurement results might vary and have introduced bias in reported risk estimates. Moreover, although SYN-JEM estimates were modelled based on silica measurements obtained during a time span of several decades, no measurements were available before 1960. We therefore assumed a constant exposure time trend prior to 1960 (10) regarding all SYN-JEM specifications, making study participants’ exposure estimates prior to 1960 possibly less accurate. Still, exposure estimates generated using alternative time trends in SYN-JEM have shown to be highly correlated (13).

### Concluding remarks

The established exposure–response relationship between occupational silica exposure and lung cancer in pooled case–control studies is marginally influenced by the exclusion of specific dimensions of SYN-JEM, which underscores the quantitative JEM as a methodological flexible and viable exposure assessment tool in community-based studies. For optimal modelling of exposure–response relationships between occupational exposures and health risk we recommend incorporating all relevant dimensions (ie, prior rating, job, time and region), with a particular emphasis on the inclusion of quantitative job-specific estimates which appeared to be the most prominent for this general population JEM.

## Supplementary material

Supplementary material
