# Supplementary material for: Respirable crystalline silica and lung cancer in community-based studies: impact of job-exposure matrix specifications on exposure–response relationships
Source: Scand J Work Environ Health. 2024 Mar 27;50(3):178–86. doi: 10.5271/sjweh.4140 (PMC11064806; doi:10.5271/sjweh.4140)
Supplement: Supplementary material [file SJWEH-50-178-S001.pdf]

Respirable crystalline silica and lung cancer in community-based studies: impact of job-exposure matrix specifications on exposure–response relationships[1]

by Johan Ohlander,[2] Hans Kromhout Roel Vermeulen, Lützen Portengen. Benjamin Kendzia, Barbara Savary, Domenico Cavallo, Andrea Cattaneo, Enrica Migliori, Lorenzo Richiardi, Nils Plato, Heinz-Erich Wichmann, Stefan Karrasch, Dario Consonni, Maria Teresa Landi, Neil E Caporaso, Jack Siemiatycki, Per Gustavsson, Karl-Heinz Jöckel, Wolfgang Ahrens, Hermann Pohlabeln, Guillermo Fernández-Tardón, David Zaridze, Jolanta Lissowska, Beata Swiatkowska, John K Field, John R McLauglin, Paul A Demers, Tamas Pandics, Francesco Forastiere, Eleonora Fabianova, Miriam Schejbalova, Lenka Fretova, Vladimir Janout, Dana Mates, Christine Barul, Thomas Brüning, Thomas Behrens, Kurt Straif, Joachim Schüz, Ann Olsson, Susan Peters

1. Supplementary material
2. Correspondence to: Johan Ohlander, Institute for Risk Assessment Sciences, Utrecht University, Utrecht, the Netherlands. [E-mail: j.p.g.ohlander@uu.nl]

File 1

SYN-JEM and alternative SYN-JEM specifications

Levels of no, low or high silica exposure were assigned to all ISCO 1968 job-codes by DOM-JEM, which was used as a prior in the statistical model to create SYN-JEM. Based on measurements collated in the database ExpoSyn [1], a mixed-effects model was elaborated, resulting in estimates for region ($Random_{region}$; Canada, France, Germany, the UK, and Northern, Southern, and Western Europe), job title ($Random_{job}$; for ISCO codes with exposure measurements available), year ($ß_{year}$; -6% per year), DOM-JEM score ($ß_{jem\ score}$; no, low or high exposure), and sampling duration ($ß_{sampling\ duration}$*480 minutes). The latter was applied to predict the exposure level for an eight-hour work-shift. All estimates were standardized to a representative work situation. The expression used for the predictions in the quantitative SYN-JEM is given below (all silica levels were in mg/m^3):

$$Ln(Y) = ß_0 + ß_{jem\ score} + Random_{job} + Random_{region} + ß_{year} + (ß_{sampling\ duration} * 480\ minutes)$$

SYN-JEM **(JEM 1)** consists of three axes: job, region, and year. The exponent of the natural log-transformed exposure level (ln(Y)) provides an annual geometric mean exposure level to silica for a job-region-year combination. The elaboration of SYN-JEM and the tested alternatives are briefly described below.

Region - Estimates for all regions in the SYNERGY population could be derived from the statistical model, except for the Central and Eastern European (CEE) countries due to absence of measurement data. For this region, which was assumed to be relatively high exposed, the highest region/country estimate was assigned (i.e. UK). The effect of not assigning the country/region effect to SYN-JEM (i.e. no difference in job estimates by country/region) was explored as alternative **JEM 2**.

Job estimate - For jobs classified as exposed by the DOM-JEM and with exposure measurements available, we assigned a job-specific estimate. We set an arbitrary minimum of five measurements per job title to reduce the influence of sampling error. If there were fewer or no data points for a job, the estimate from a similar job was applied. In ExpoSyn fewer than five measurements were available for the following job titles (ISCO-68 job codes): 61230, 62105, 71120, 89440, 89950, 89960, 95130, 95930, 62220, 71140, 72440, 95150, 95155, 97425, 98360, 62330, 95900, 89970. The most comparable job was manually assigned based on the ISCO job description and DOM-JEM score. We tested the influence on SYN-JEM exposure estimates of not assigning the job-specific estimate, but assigning only job estimates based on the DOM-JEM rating **(JEM 3)**. Additionally, an alternative on SYN-JEM exposure estimates without assignment of region- and job-specific estimates was tested **(JEM 4)**.

Time trend - In our model we estimated an overall linear time trend of -6% per year. This time trend was applied for the period from 1960 onwards. An exposure ceiling was assumed for the years before 1960; assigning the 1960-estimate to all previous years.

DOM-JEM prior - Jobs classified as nonexposed by the DOM-JEM were assigned an exposure level of 0 mg/m^3 as an override. The use of the DOM-JEM as a prior in the statistical model allowed also for calibration of exposure levels by a weighted mean of exposure measurements. An exposure level for all potential jobs can be calculated using these estimates for low and high exposed jobs, even in the absence of measurement data. The statistical model provided job-specific estimates for each job with measurement data, hence also for jobs considered nonexposed (according to DOM-JEM). However, those measurements are likely to represent only exceptional situations where exposures occur. As an alternative we tested the model without application of the DOM-JEM prior albeit with the nonexposed override **(JEM 5)**. Any job without measurement data was assumed nonexposed in these alternatives.

Figures S1-S4. Exposure-response curves for cumulative silica exposure in relation to lung cancer risk based on general additive models (GAM), as per the original SYN-JEM (JEM 1) compared with each analyzed SYN-JEM specifications with varying dimensions of SYN-JEM included (JEMs 2-5). 95% confidence intervals are marked with light grey shade for JEMs2-5 and with dark grey shade for JEM 1.

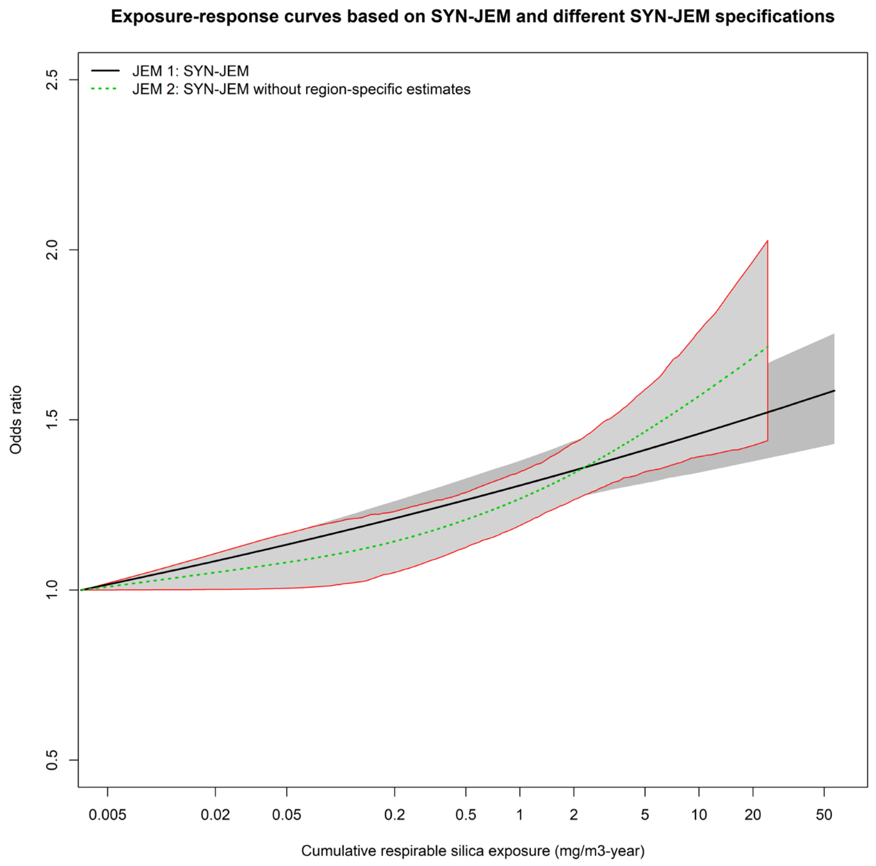
Exposure-response curves based on SYN-JEM and different SYN-JEM specifications
JEM 1: SYN-JEM
JEM 2: SYN-JEM without region-specific estimates
Odds ratio
0.5
1.0
1.5
2.0
2.5
0.005
0.02
0.05
0.2
0.5
1
2
5
10
20
50
Cumulative respirable silica exposure (mg/m3-year)

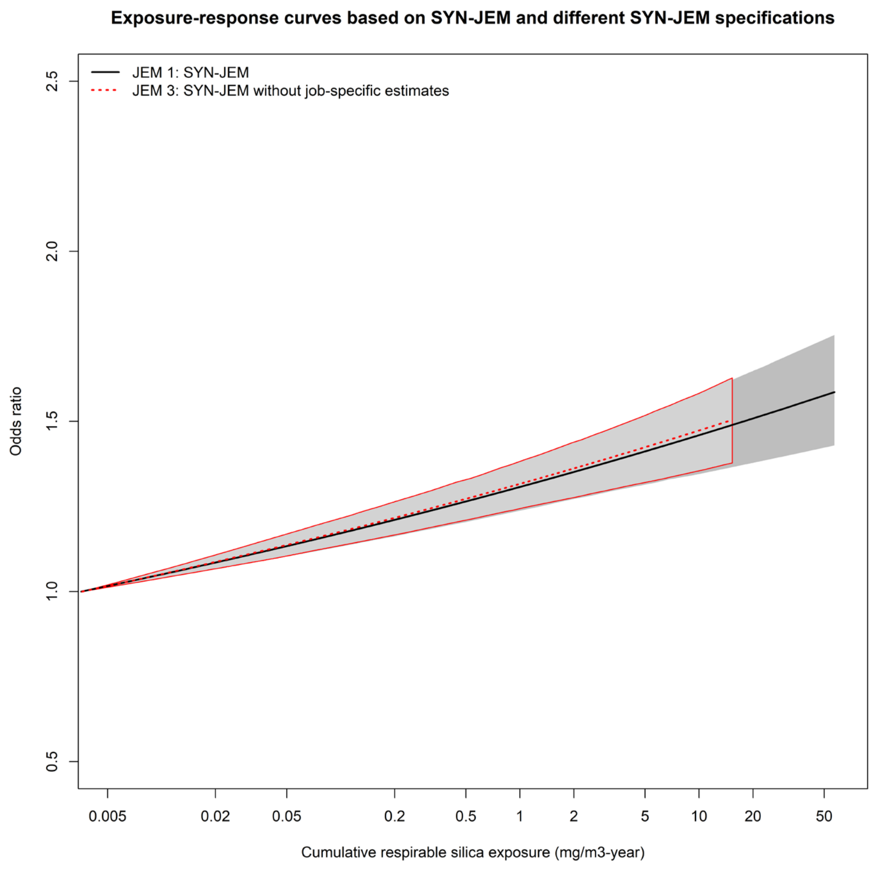
Exposure-response curves based on SYN-JEM and different SYN-JEM specifications
JEM 1: SYN-JEM
JEM 3: SYN-JEM without job-specific estimates
Odds ratio
0.5
1.0
1.5
2.0
2.5
0.005
0.02
0.05
0.2
0.5
1
2
5
10
20
50
Cumulative respirable silica exposure (mg/m3-year)

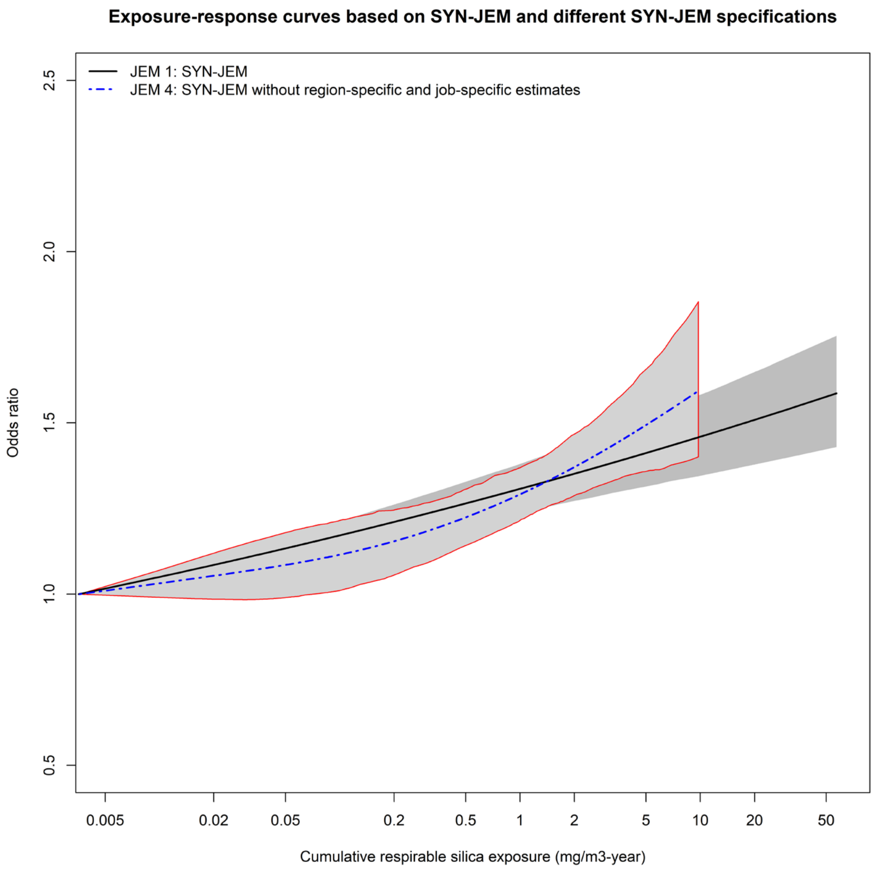
Exposure-response curves based on SYN-JEM and different SYN-JEM specifications
JEM 1: SYN-JEM
JEM 4: SYN-JEM without region-specific and job-specific estimates
Odds ratio
0.5
1.0
1.5
2.0
2.5
0.005
0.02
0.05
0.2
0.5
1
2
5
10
20
50
Cumulative respirable silica exposure (mg/m3-year)

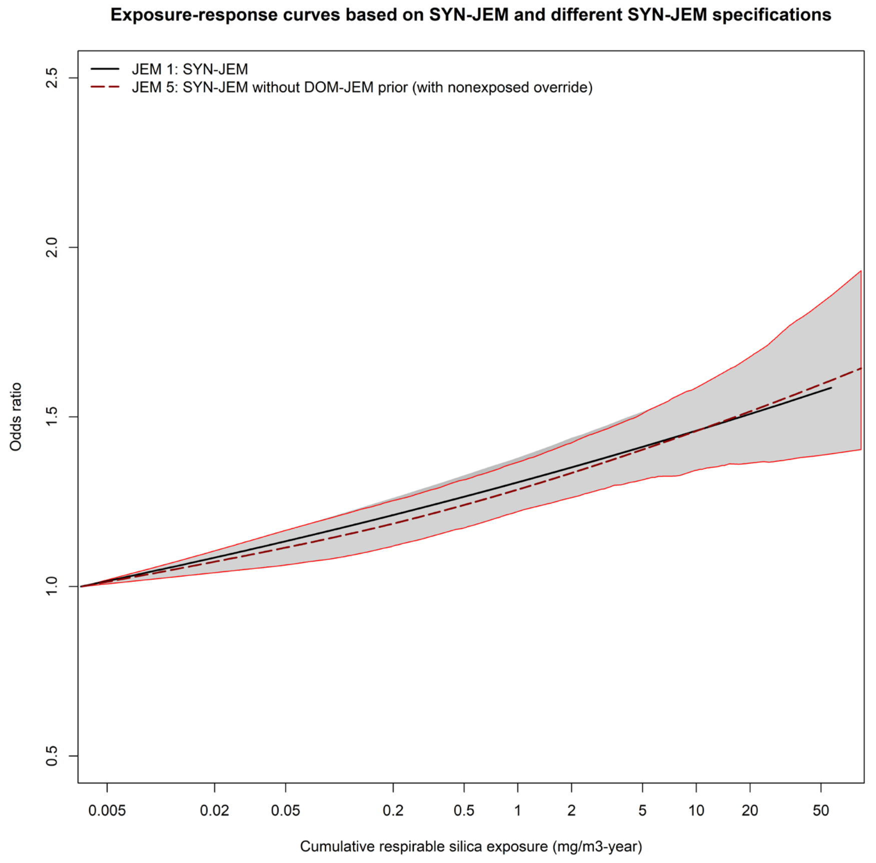
Exposure-response curves based on SYN-JEM and different SYN-JEM specifications
JEM 1: SYN-JEM
JEM 5: SYN-JEM without DOM-JEM prior (with nonexposed override)
Odds ratio
0.5
1.0
1.5
2.0
2.5
0.005
0.02
0.05
0.2
0.5
1
2
5
10
20
50
Cumulative respirable silica exposure (mg/m3-year)

References for Supplementary Material

1. Peters S, Vermeulen R, Olsson A, Van Gelder R, Kendzia B, Vincent R, et al. Development of an exposure measurement database on five lung carcinogens (ExpoSYN) for quantitative retrospective occupational exposure assessment. Ann Occup Hyg. 2012;56:70-9.
